# The Voluntary Hospitals Point the Way

**Published:** 1913-01-11

**Authors:** 


### The Hospital

The Workers' Newspaper of Administrative Medicine and Institutional Life, Administration, National Insurance and Health.

No. 1384, Vol. LIII. SATURDAY, JANUARY 11, 1913. PRICE ONE PENNY.

### THE VOLUNTARY HOSPITALS POINT THE WAY.

The voluntary hospitals have been placed in no little difficulty owing to the National Insurance Act, which comes into force upon the 15th instant. This position could never have arisen had there been an exhibition of adequate statesmanship and care in the elaboration of a great scheme of national insurance which everybody is in favour of and which all would welcome. The whole of the difficulties are due to unjustifiable and unnecessary haste, and to reckless indifference to those business methods which ought to characterise every official act of the Government of a great Empire. The hospitals may have gathered some consolation from the intimation of the Chancellor on December 17 "that the question of hospitals had been by no means overlooked in planning the Government's Insurance scheme, but that it had been deemed wiser to endeavour to avoid any steps that would imperil the voluntary nature of the great hospitals of this country. This could not have been avoided if hospital work had been brought into the scheme of the Act, since a subsidy from public funds would necessarily have been followed by some degree of public control." We have shown in these coumns over and over again during the past few months how an exhibition of wise statesmanship could and ought to have brought the voluntary hospitals into the scheme of national insurance, not by subsidy, but by a plan of co-operation which would have overcome the bogey of public control set up by the Chancellor of the Exchequer. We have asked the Chancellor to define what he means by public control, and are awaiting his official reply. If he means public hospitals to the elimination of the voluntary hospitals, he must be prepared to provide an additional annual revenue of £20,000,000, as was brought out by the Poor Law Commission. We may surely conclude, therefore, that he does not mean State hospitals. If he means Government inspection of all wards occupied by insured persons, the hollowness of the bogey raised by Mr. Lloyd George is strangled from its birth. There is therefore no reason except hurry and absence of statesmanship to justify the risk of permanently and unnecessarily endangering by legislation the revenue of our voluntary hospitals as they exist to-day.

The voluntary hospital managers have shown great public spirit, and have raised themselves immeasurably in public estimation, by accepting the position and agreeing to make the best of it during the present year, so that the precise effect of National Insurance may be made clear. They are fully conscious that the medical treatment afforded to insured persons under the Act will be such treatment as can properly be given by a general practitioner of ordinary competence and skill, and this is just the kind of treatment that the hospital does not exist to give. Accepting this definition, the voluntary hospitals will continue the treatment of the really necessitous poor so far as they may be eligible for hospital benefit. Every person attending a voluntary hospital on and from the 15th instant will be registered, being an insured person. Their treatment, and the class of treatment they will receive at the voluntary hospitals, will be governed by the nature and urgency of the malady or injury from which they may be suffering. In the case of out-patients the general practice will be that, except for accidents, emergencies, or such special treatment as can only be given in a hospital, insured persons will no longer be received in the out-patient and casualty departments unless accompanied by a certificate or introduced personally by the medical practitioner who is in attendance. With reference to in-patients, insured persons whose cases are urgent and in need of hospital treatment not provided for under the National Insurance Act will be admitted as heretofore, but an accurate record of every person so admitted and of the approved society to which he may belong will be kept. In this way insured persons, despite the omissions and defects of the National Insurance Act, will have to the utmost extent possible the protection and succour of the voluntary hospitals. It is foreseen, however, that the effect of the panel system of medical relief imposed upon some 15,000,000 of people must in all probability cause a great increase in the number of persons who will apply at the hospitals for relief during the present year at any rate. If this forecast prove to be correct, it will soon become apparent that a much larger number of hospital beds than is at present available will be needed, or that insured persons must suffer for the want of adequate and immediate hospital treatment at the very moment when they need it most.

The outlook is full of interest, but if the Government frankly recognise the mistake into which they have fallen by ignoring altogether the most expensive form of treatment for insured persons, *i.e.* hospital treatment, they will wisely set up, under the National Health Insurance Commission, simple but adequate machinery controlled by expert knowledge to watch the hospital problem. Such machinery would gather information and so be prepared to meet any emergency which may arise, and thus in the end and out of the muddle all might ultimately work out even yet to the satisfaction and benefit of the people and their voluntary hospitals. The voluntary hospitals must in fact prove, as the Act stands at present, the financial strength or weakness of the approved societies set up under the National Insurance Act. This is so because, unless the hospital treatment absolutely essential to the speedy recovery of insured persons, when overtaken by accident or attacked by disease, is forthcoming as and when it is required, the sick pay demands which the approved societies will have to meet must speedily exhaust the whole of the funds available and untimately undermine their financial efficiency to the breaking point. The voluntary hospital managers have done their duty by patriotically stepping into the breach. The Government, and especially Mr. Lloyd George as Chancellor of the Exchequer, must, however, do their duty forthwith by making provisional arrangements whereby it will be possible, should the hospitals be crowded out with insured persons needing hospital treatment, to approach the voluntary hospital managers and ask them to co-operate with them, on a *pro rata* basis and a system of loans for new hospital buildings. This basis and the system of loans, as we have fully explained in former articles, would enable adequate accommodation through the voluntary hospitals to be provided without delay, so that every insured person may receive the treatment essential to his condition, and that the approved societies may be protected from the financial ruin which, in the absence of such hospital accommodation, must speedily overtake the majority of them.

